# A Comprehensive Review of Thoracic Aortic Disease in Immunosuppressed States: Clinical Signals, Mechanisms, and Implications for Surveillance

**DOI:** 10.3390/jcdd13060224

**Published:** 2026-05-25

**Authors:** Yashraj Srivastava, Korri Hershenhouse, Isaac Faith, Tanner Nelson, Brandon E. Ferrell, Ahren J. Alberto, Tadahisa Sugiura

**Affiliations:** 1 Albert Einstein College of Medicine, Bronx, NY 10461, USA; yashraj.srivastava@einsteinmed.edu (Y.S.);; 2 Department of Cardiothoracic and Vascular Surgery, Montefiore-Einstein Medical Center, Bronx, NY 10467, USA

**Keywords:** aortic aneurysm, thoracic aortic aneurysm, thoracic aortic dilation, immunosuppression, malignancy, autoimmune disease, organ transplant, HIV

## Abstract

Background: Immune dysregulation and clinical immunosuppression are biologically plausible contributors to thoracic aortic wall vulnerability through endothelial injury, protease-mediated extracellular matrix remodeling, vascular smooth muscle cell dysfunction, and impaired vascular repair. Yet, the clinical relevance of immunomodulated states to thoracic aortic aneurysm (TAA) incidence or growth and acute aortic syndromes remains undefined. Methods: This comprehensive review synthesizes clinical and translation evidence linking immunomodulated states in solid organ transplantation, autoimmune disease (predominantly systemic lupus erythematosus), HIV, and oncologic therapies to thoracic aortic dilation, aneurysmal progression, and acute aortic events. Principal Findings: Across transplant, autoimmune, and HIV cohorts, recurring themes include chronic immune dysregulation, endothelial dysfunction, proteolytic matrix remodeling, and impaired vascular repair capacity, although thoracic segment-specific longitudinal growth data remain limited and are often embedded within analyses of multiple vascular beds. In oncologic cohorts, aggregate analyses generally do not demonstrate uniform acceleration of aneurysm growth with malignancy or chemotherapy exposure, although agent-level models suggest that regimen-specific effects may be obscured in pooled estimates. Two studies most directly addressed our question in thoracic-relevant contexts reported (1) very low mean annual ascending aortic aneurysm growth (0.18 ± 0.64 mm/year) with no detectable association with chemotherapy or radiotherapy and (2) prior immunosuppressive/cytostatic chemotherapy exposure to be common in a proximal TAA surgical cohort (39.3%) without a clear difference in thoracic phenotype at presentation or postoperative outcomes. In HIV cohorts, available evidence supports modest but reproducible proximal aortic remodeling and a clinically meaningful aneurysm burden across vascular beds, yet definitive thoracic segment-specific natural history data remain limited. Conclusions: The available literature supports clinical vigilance and exposure-aware surveillance, while suggesting that thoracic aortic risk is unlikely to be uniform across immunosuppressive and cytotoxic therapies. Standardized, segment-specific longitudinal imaging with granular agent-level exposure characterization (dose, duration, sequencing, and combination regimens), consistent definitions of baseline diameter and growth, careful adjustment for key confounders, and prospective ascertainment of dissection/rupture and operative endpoints are needed to translate immunobiology into actionable risk stratification and long-term management strategies.

## 1. Introduction

Thoracic aortic aneurysm (TAA) is an insidious disease process that is often asymptomatic until dissection or rupture. In a meta-analysis of population-based studies, the pooled incidence of TAA was 5.3 per 100,000 persons/year [1]. TAAs warrant structured surveillance, as acute aortic events occur increasingly with increasing diameter [2,3].

The 2022 American Heart Association guidelines recommend surveillance for TAAs at varied intervals from 6 to 24 months based on aortic stability and patient risk factors. Guideline-directed elective repair thresholds are determined by a constellation of factors. In the modern era, aortic diameter is only one component of contemporary risk stratification. Decisions to intervene should incorporate aneurysm growth rate, aortic length, patient stature, family history of aortopathy and sudden death, syndromic disease, bicuspid aortic valve (BAV), and patient comorbidities and operative risk [3,4,5]. Decisions to intervene should incorporate aneurysm growth rate, aortic length, patient stature, family history of aortopathy and sudden death, syndromic disease, bicuspid aortic valve (BAV), and patient comorbidities and operative risk [3,4,5]. Guidelines do remain in place supporting traditional intervention thresholds such as 4.5 cm in hereditary, syndromic TAA, 5.0 cm for BAV or aortic root aneurysms, and rapid growth (≥0.3 cm/year over 2 consecutive years or ≥0.5 cm in 1 year). However, they have been recently updated to reflect earlier intervention thresholds (5.0 cm, Class IIa) for ascending TAA at an experienced aortic center, taking into account aortic length, family history, and patient age [3,5].

The movement toward intervention at smaller diameters and multifaceted patient risk assessments stems from findings that 40% of patients present with dissection prior to meeting traditional aneurysm diameter thresholds (5.5 cm) for elective intervention [6]. Therefore, it remains crucial to identify other clinical factors placing patients at risk for aortic growth and rupture.

An acute aortic syndrome (AAS) is an acute disruption of aortic wall integrity that may result in an aortic dissection (80%) from an intimal tear with creation of a false lumen, intramural hematoma (15%) from hemorrhagic rupture of the medial vasa vasorum, or penetrating aortic ulcer (5%) from ulceration of an atherosclerotic plaque extending through the internal elastic lamina into the medial layer [4]. Clinically, the Stanford classification is used to describe aortic dissection, with type A involving the ascending aorta and type B involving aortic segments distal to the innominate artery [4,5]. Acute dissection and rupture represent the end-stage of aortic aneurysmal disease. Given the high mortality of these conditions, close surveillance and appropriate identification of aneurysm patients at high risk remains crucial.

The evolution of TAA is a complex interplay between chronic mechanical stress and elastic medial degeneration. Key mechanisms for aneurysm development include vascular smooth muscle cell dysfunction and phenotypic switching from a contractile phenotype to a synthetic, pro-inflammatory/migratory phenotype. Negative remodeling of the extracellular matrix (ECM), elastin fragmentation, and maladaptive signaling networks (e.g., dysregulated transforming growth factor-β (TGF-β)/SMAD signaling, angiotensin II-mediated pathways, mitogen-activated protein kinase (MAPK) cascades (e.g., ERK/p38/JNK), and nuclear factor-κB (NF-κB)-associated inflammatory signaling) erode integrity of the aortic media [7]. This process differs from the development of abdominal aortic aneurysms (AAAs), which are more strongly associated with atherosclerotic risk and plaque-prone infrarenal aortic disease. In AAA, intimal lipid deposition and degenerative calcification contribute to disturbed flow with oscillatory wall shear stress and regionally increased wall stress, promoting local inflammation and proteolytic remodeling [8]. Inflammatory activity resulting in immune cell recruitment, protease activity, and ECM destabilization is actively being studied for its role in aneurysm formation and expansion [9]. Within this model, the role of adaptive immunity in aortic wall destabilization and disease propagation remains incompletely mapped [10]. Immune alterations are seldom discussed in the cardiothoracic literature as contributors to aortic wall weakening, expansion, and rupture.

Linking clinical evidence on TAA and acute aortic events (AAEs) in immunomodulated states could refine risk profiling and surveillance strategies for patients receiving immunosuppressive or cytotoxic therapies. Here, we provide a comprehensive synthesis of evidence on TAAs and AAEs across transplant surgery, malignancy, autoimmune disease, and acquired immunodeficiency.

## 2. Scope and Evidence Identification

We identified relevant studies through targeted PubMed/MEDLINE searches spanning 1 January 2000 to 1 January 2026, supplemented by citation review of seminal papers. We prioritized adult human studies reporting thoracic segment outcomes or proximal aortic measurements in the context of transplant immunosuppression, autoimmune disease, HIV, and oncologic therapies. Syndromic/heritable aortopathies and primary large vessel vasculitides were considered outside the scope of this review. Given heterogeneity in exposure definitions and outcome ascertainment, evidence was synthesized narratively with an emphasis on mechanistic plausibility, thoracic segment specificity, and clinical interpretability.

## 3. Evidence Synthesis

The available literature addressing thoracic aortic pathology in immunomodulated states is heterogeneous in design and clinical context, spanning retrospective surgical cohorts, single-center observational imaging studies, database analyses, and cohort studies across transplant recipients, patients with malignancy receiving oncologic therapies, autoimmune disease populations (predominantly systemic lupus erythematosus), and people living with HIV (PLWH). Table 1 summarizes the study design, patient population, sample size, exposure definitions, aortic segments assessed, endpoint definitions, comparator groups, and follow-up from the studies captured by our literature search. Because exposure definitions and thoracic segment ascertainment varied substantially across studies, findings are summarized thematically with emphasis on thoracic relevance and mechanistic plausibility.

## 4. Immunosuppression in Transplant Cohorts

Across transplant cohorts, the clinical signal is most consistent with a shift toward impaired vascular repair capacity under chronic immunosuppression, with limited thoracic-specific longitudinal growth data. In the transplant-focused literature, shifts in aortic wall remodeling in the setting of chronic immunosuppression have been attributed to altered protease balance (e.g., MMP-2/MMP-9 and TIMPs), endothelial dysfunction, and impaired vascular smooth muscle cell proliferation and migration, which may blunt adaptive repair under chronic hemodynamic stress [9,10,26,27]. Reported immunosuppressive exposures include glucocorticoids, calcineurin inhibitors (tacrolimus/cyclosporine), antimetabolites (e.g., mycophenolate or azathioprine), mTOR inhibitors (sirolimus/everolimus), and antineoplastic (i.e., cytostatic) chemotherapy in one proximal TAA surgical cohort [11,21,27]. However, few published studies pair granular exposure phenotyping with thoracic aortic histopathology, serial inflammatory/proteolytic biomarkers, or standardized longitudinal thoracic imaging.

Three representative transplant-related datasets illustrate how current thoracic inference is derived: (i) a proximal TAA surgical cohort assessing prior immunosuppressive/cytostatic exposure [11], (ii) a kidney transplant echocardiographic study comparing aortic root dimensions by maintenance regimen (mTOR inhibitor vs. calcineurin inhibitor) [21], and (iii) a post-abdominal organ transplant aneurysm cohort with thoracic aneurysm reporting [22].

In a proximal surgical TAA cohort, Ostovar et al. retrospectively analyzed 224 patients undergoing proximal aortic aneurysm surgery and identified prior immunosuppressive and/or cytostatic exposure in 88 (39.3%) [11]. Exposure categories included glucocorticoids (*n* = 43), chemotherapy (*n* = 49), and other non-glucocorticoid immunosuppressive agents (*n* = 13), with overlap between categories; common indications included rheumatoid arthritis, polymyalgia rheumatica, giant cell arteritis, and prior malignancy. The authors did not report disease activity, cumulative dose, treatment duration, or latency from exposure initiation to diagnosis/operation. Short- and long-term postoperative outcomes and survival were comparable between exposure groups, and the authors speculated that immune dysregulation and impaired inflammatory cell trafficking may contribute to aneurysm development in susceptible patients.

In a kidney transplant echocardiography cohort, Obremska et al. compared 87 recipients on mTOR inhibitor versus calcineurin inhibitor regimens and found larger aortic root diameters (37.1 ± 4.9 vs. 34.5 ± 3.5 mm, *p* = 0.01) and ascending aortic diameters (36.8 ± 5.1 vs. 34.4 ± 3.1 mm, *p* = 0.03) in the mTOR inhibitor group [22]. In regression models, they evaluated associations with age, sex, body surface area, time after transplantation, diabetes, blood pressure, lipids, and renal function; in stepwise analyses, aortic root diameter remained associated with body surface area and mTOR inhibitor exposure. The authors hypothesized that mTOR inhibition may impair endothelial and vascular smooth muscle cell proliferation and migration required for vascular repair and regeneration, thereby predisposing susceptible recipients to dilation.

In an abdominal organ transplant cohort, Cron et al. reviewed 127 transplant recipients with aneurysms, including 22 thoracic aortic aneurysms, and reported that most aneurysms (83/127) were first identified after transplantation, while all documented aortic ruptures occurred in the post-transplant period [21]. Aneurysm distribution varied by transplanted organ (e.g., kidney transplant recipients most commonly had AAA, whereas liver transplant recipients more often had visceral artery aneurysms). In the subset with serial measurements, they observed a higher mean AAA expansion rate after transplantation (0.58 ± 0.48 vs. 0.41 ± 0.16 cm/year pre-transplant), but thoracic growth analyses were limited by sample size and scarce longitudinal imaging. The authors hypothesized that chronic steroid exposure and post-transplant hypertension/pulsatile stress could contribute to aneurysm progression. Overall, the transplant literature supports biologic plausibility for altered balance of repair versus injury under immunosuppression, but standardized thoracic segment-specific surveillance and growth characterization remain key gaps.

## 5. Oncologic Therapy Exposure Cohorts

Across oncologic cohorts, pooled analyses generally do not demonstrate uniform acceleration of aneurysm growth with malignancy or chemotherapy exposure, although regimen-level signals emerge in selected models and may be diluted when therapies are aggregated. Proposed contributors to aneurysm progression include treatment-induced hemodynamic stress, endothelial injury, oxidative stress, impaired collagen and elastin synthesis, smooth muscle cell injury, and proteolytic remodeling (including MMP-2/MMP-9 activity) [9,12,23,24,25,27,28,29,30].

Existing oncology studies are heterogeneous in aneurysm segment, regimen exposure, and study design. In a small single-center series of 19 patients with concomitant malignancy and aortic aneurysms, Leopardi et al. reported no aneurysm growth among patients not receiving oncologic therapy, whereas those receiving active treatment (including one TAA) demonstrated mean sac growth of 2.9 cm over 6 months and two urgent repairs for rupture [12]. Although the authors reported the administered agents, temporal/dose relationships were not specified and multi-agent exposure limited attribution to a single drug. In contrast, Martin et al. examined 91 patients receiving cytotoxic chemotherapy with coexisting aortic aneurysms across vascular segments and found growth rates similar to non-chemotherapy surveillance controls (2.3 vs. 2.4 mm/year), despite frequent multi-agent exposure and substantial concomitant steroid use (84/91, 92%) [24]. In a larger retrospective cohort (159 patients with malignancy/172 aneurysms vs. 127 patients without malignancy/149 aneurysms), Maxwell et al. also found similar median growth rates overall (0.12 cm/year) and fewer ruptures in the malignancy cohort; however, class-specific multivariable analyses identified antimetabolite exposure (*n* = 51) as independently associated with higher growth (+0.17 cm/year, 95% CI 0.074–0.266; *p* = 0.001), while anti-VEGF exposure (*n* = 15) was not associated with a different growth rate (0.26 vs. 0.27 cm/year; *p* = 0.898) [23]. Taken together, these data argue against a uniform chemotherapy-associated acceleration effect at the cohort level while suggesting regimen-specific vascular toxicity may be obscured in presently available pooled estimates.

Importantly, thoracic segment context may modify these relationships. In an ascending aorta specific (AscAA) malignancy cohort, Becker von Rose et al. evaluated 151 patients (145 analyzable for growth after excluding six with baseline AscAA > 55 mm) and reported a low mean annual AscAA growth rate of 0.18 ± 0.64 mm/year [25]. Neither tumor entity nor chemotherapy or radiotherapy alone or in combination was significantly associated with altered AscAA growth, supporting that ascending aortic remodeling may not mirror reported AAA behavior across heterogeneous oncologic regimens. Overall, oncology-associated aneurysm progression appears inconsistent in aggregate, with the most actionable signal being the missing agent-level exposure reporting and thoracic segment-specific analyses when attempting to translate vascular toxicity paradigms to the ascending aorta.

## 6. Autoimmune Disease Cohorts

Across autoimmune cohorts (predominantly SLE after excluding primary vasculitides), the most consistent clinical findings were elevated aneurysm/dissection risk in a duration-dependent fashion, more compatible with cumulative vascular injury rather than a purely acute inflammatory mechanism. Proposed mechanisms include chronic immune-mediated vascular injury (immune complex/complement activation, cytokine-driven endothelial dysfunction, and ECM remodeling), accelerated atherosclerosis, and corticosteroid-associated connective tissue fragility. Inflammatory cytokine signaling can promote oxidative stress, vascular smooth muscle cell phenotypic switching (contractile to migratory/proliferative), and upregulation of proteases (including MMP pathways) that degrade elastin/collagen. Meanwhile, chronic glucocorticoid exposure may impair collagen synthesis and tissue repair and promote hypertension, providing a plausible route to aneurysm formation, progression, or dissection in susceptible patients [7,9,14,29,30,31,32].

In a nationwide Taiwanese database study, Wang et al. compared 15,209 patients with SLE to matched healthy controls and identified 20 aortic aneurysms plus 13 aortic dissections in the SLE cohort (4.26 per 10,000 person-years), with a significantly higher incidence rate ratio for aneurysm/dissection (IRR 3.34, 95% CI 1.71–6.91) [14]. In multivariable analyses, age, male sex, hypertension, and longer SLE duration were associated with aneurysm/dissection, supporting a cumulative vascular injury model. In a single-center Chinese SLE cohort, Zhang et al. reported 16/1843 (0.86%) aortic aneurysms, predominantly abdominal (12/16), with TAAs comprising 4/16 [20]. Longer SLE duration and anti-RNP positivity were independently associated with aneurysm occurrence on multivariable analysis. In outcome analyses, 14/160 (8.75%) SLE patients without aneurysm died over a median follow-up of 10 years, whereas survival was worse among aneurysm patients with follow-up (3/14 [21.4%] deaths), including one rupture without surgery occurring 3 years after diagnosis and two postoperative deaths (timing not specified). Kurata et al. provide a useful interpretive framework by proposing two broad patterns: an inflammatory phenotype associated with active SLE-induced vasculitic injury and cystic medial degeneration versus a chronic aneurysmal/atherosclerotic phenotype associated with long-term corticosteroid exposure and cumulative vascular injury [19]. Overall, the SLE literature supports both a duration-dependent cumulative risk signal and a smaller subset of inflammatory/aortitis presentations, with thoracic segment specificity varying across study designs.

## 7. Acquired Immunodeficiency

Across HIV cohorts, the most consistent findings are a modest increase in proximal aortic remodeling and a clinically meaningful aneurysm burden across vascular beds. Proposed mechanisms are increasingly supported by imaging and biomarker studies, demonstrating persistent immune activation (even on HAART), endothelial dysfunction, dysregulated cytokine signaling (including TNF-α and IL-6 pathways), oxidative stress, and protease-mediated matrix degradation. These processes result in accelerated atherosclerosis, and vasculitic or vasa vasorum-related injury as contributors to aneurysm formation and growth [13,15,17,18,33]. Within this framework, HIV has been associated with increased aneurysm prevalence on CT, larger proximal aortic dimensions on echocardiography, and elevated biomarkers of platelet activation, thrombo-inflammatory activation, and endothelial disruption in cohorts of PLWH [13,19,33].

Available HIV studies include CT-based imaging cohorts, echocardiographic dimensional analyses, surgical case series, and national inpatient administrative data [13,15,16,17,18]. Høgh et al. performed a prospective matched cohort study in Denmark (594 PLWH aged ≥ 40 years vs. 1188 matched controls) using contrast-enhanced CT to assess aortic dimensions and define aneurysms per European Society of Cardiology criteria [13]. They found aneurysms in 42 PLWH (7.1%) versus 29 controls (2.4%). HIV status was independently associated with aneurysm presence (aOR 4.51, 95% CI 2.56–8.08).

Using echocardiographic data from the Multicenter AIDS Cohort Study, Minhas et al. found that HIV infection (*n* = 1164) was associated with modest increases in aortic root diameter (0.03 cm, 95% CI 0.002–0.06 cm) and ascending aortic diameter (0.04 cm, 95% CI 0.01–0.06 cm) compared with uninfected participants [15]. Specifically among men with HIV, lower nadir CD4+ counts and higher TNF-α levels were associated with larger proximal aortic dimensions, supporting a cumulative immune injury hypothesis. Grønbæk et al. further linked aneurysm presence in PLWH to elevated biomarkers of platelet activation and procoagulant activity. They reported independent associations of elevated sCD40L (aOR 2.67) and D-dimer (aOR 1.90) with HIV-associated aneurysm presence [17].

Clinically, surgical series suggest meaningful disease burden in selected populations. Kim et al. retrospectively reviewed 104 PLWH with 129 aneurysms across two institutions, reporting predominance of large-vessel aneurysms including 53 ascending aortic (41.1%) and 13 descending thoracic (10.1%) aneurysms [18]. Aneurysms (23/104 [22.1%)]) were noted in multiple vascular beds with 10 (9.6%) having saccular morphology. Among the 26 patients (25.0%) undergoing repair, perioperative complications occurred in 46.2%, while mortality was low (3.8%). Overall, the HIV literature supports aortic remodeling and multi-focal aneurysm burden with plausible inflammatory and endothelial mechanisms, while thoracic segment-specific growth and clinical trajectories remain an important evidence gap.

## 8. Discussion

### 8.1. Principal Findings

Our comprehensive review suggests a plausible association between chronically immunosuppressed states and thoracic aortic pathology; however, data remains heterogeneous across underlying immunomodulatory etiologies. Across transplant, SLE, and HIV cohorts, the most consistent themes are chronic immune dysregulation, endothelial injury, proteolytic ECM remodeling, and impaired vascular repair rather than a single exposure-specific pathway. Within transplant and HIV cohorts, aneurysms are reported across multiple vascular beds not uniformly including thoracic involvement. Proposed drivers in transplant cohorts emphasize cumulative vascular injury secondary to multifaceted immunosuppressive therapies (e.g., chronic steroid/immunosuppression exposure, hypertension, and impaired medial/ECM repair capacity) rather than a discrete agent-linked phenotype. In HIV cohorts, the literature most consistently supports aortic remodeling in the setting of persistent immune activation, endothelial dysfunction, and matrix-degrading inflammatory pathways. In multiple oncologic cohorts, aggregate analyses generally showed no clear acceleration of aneurysm growth with malignancy or chemotherapy exposure, but class-specific models identified potential regimen-level signals (e.g., antimetabolites associated with higher growth and topoisomerase inhibitors associated with earlier repair), suggesting that susceptible subgroups warrant further studies. This is consistent with the dedicated evaluation by Becker von Rose et al., which demonstrated low AscAA growth without a detectable association with chemotherapy or radiotherapy. Ostovar et al. provided demonstrative clinical data, examining prior cytostatic/immunosuppressive exposure in a TAA surgical population and found that exposure history was not associated with a clearly more advanced thoracic phenotype or worse postoperative outcomes. However, the prevalence of immunosuppression in approximately 40% of their cohort warrants further exploration. Taken together, the literature supports clinical vigilance when surveilling an immunosuppressed patient and a need for further exposure-specific data as well as improved reporting of segment-specific aneurysm progression.

### 8.2. Clinical Implications

**Figure 1:**
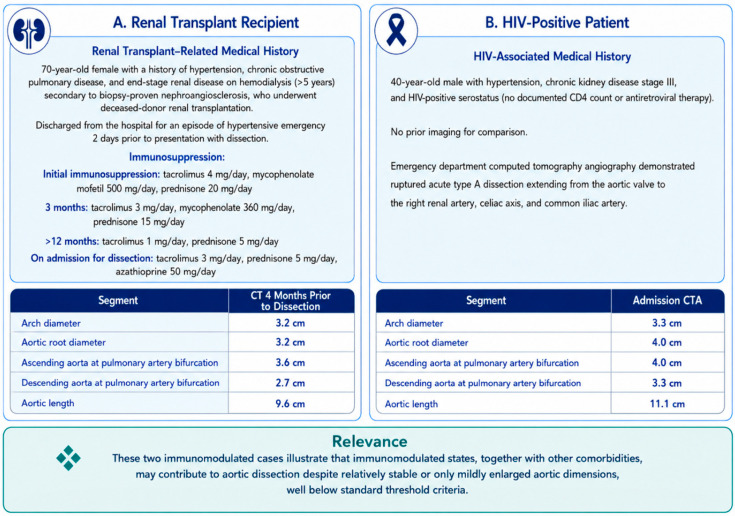
Aortic dimensions in a renal transplant patient and an HIV-positive patient with acute type A aortic dissection.

Immunosuppressive therapies are pervasive in modern clinical practice, spanning autoimmune disease, solid organ transplantation, and oncologic regimens. Potential risks posed by immunosuppressive therapy on aneurysm evolution are not currently reflected in clinical aortic intervention or surveillance guidelines. In the context of current AHA guidelines, where surveillance intensity and operative thresholds are driven by segment, diameter, and interval growth rates, our findings support further study of aortic growth rates and rupture risk in immunosuppressed patient cohorts. Closer surveillance in these patients may be warranted, as even a modest association with thoracic aortic remodeling could have an outsized clinical impact given the fatality of AAS, as illustrated by two clinical cases in Figure 1.

Contemporary population and imaging datasets in acquired immunodeficiency reinforce that HIV infection is associated with thoracic (and abdominal) aortic aneurysms, supporting the concept that immune dysregulation and chronic inflammation may influence aortic wall integrity. In contrast, observational oncologic cohorts do not show accelerated aneurysm progression in exposed patients, underscoring that effects may be heterogeneous, context-dependent, and strongly confounded by baseline risk, competing mortality, surveillance intensity, and aortic segment biology.

### 8.3. Limitations of the Existing Evidence

A major limitation of the current evidence base is the absence of standardized exposure and outcome definitions, which precludes quantitative synthesis. Exposure is commonly coded as a historical event, i.e., any immunosuppression exposure rather than being captured in a granular fashion, e.g., agent class, cumulative dose, duration, timing, and concurrent regimens, limiting any inference about dose-dependent relationships or class-specific effects. Outcomes are similarly inconsistent, i.e., some studies provide segment-level measurements (aortic root/ascending diameter) or segment-specific thoracic growth analyses, while others rely on broad aortic aneurysm constructs with limited or incomplete specification of anatomy. When paired with the small number of studies directly interrogating thoracic/ascending disease, this heterogeneity underscores that the current evidence is largely hypothesis-generating rather than decision-guiding.

### 8.4. Future Directions

Progress in this area will require prospective, anatomic segment focused, longitudinal cohorts that standardize both exposure characterization and thoracic outcome ascertainment. To enable cross-study comparability and clinically actionable inference, future work should incorporate reproducible, synthesizable reporting and design elements. Mechanism-linked categorization (circulating inflammatory/proteolytic biomarkers, immune profiling, or aortic tissue histopathology) will allow for mapping of clinical associations to plausible pathways (e.g., adaptive immunity, endothelial dysfunction, and ECM remodeling). Prospective ascertainment of thoracic segment specific outcomes, including growth, acute dissection/rupture, perioperative outcomes, and long-term survival, will allow for development of risk stratification of these patients. Routinely addressing patient factors and confounding variables, including age, sex, BMI/BSA, hypertension, smoking, lipid disease, diabetes, baseline aortic size, and surveillance frequency with transparent handling of competing mortality risks, particularly in oncology cohorts, will allow for an in depth understanding of the baseline risk of the cohort and the interaction of immunosuppressive agents with other risk factors at play. Granular agent-level exposure capture, including drug class, dose, duration, sequencing/latency, and combination regimens (including concurrent glucocorticoid burden), will allow for agent-specific impact to be assessed, and standardized, segment-specific imaging protocols with clearly defined measurement planes and modality (e.g., ECG-gated CT vs. non-gated CT vs. echocardiography) will provide reproducible definitions of baseline diameter and annualized growth.

## 9. Conclusions

Across immunomodulated states, the available literature supports a plausible relationship between thoracic aortic negative remodeling and immune dysregulation based on mechanisms of endothelial injury, protease-mediated extracellular matrix remodeling, and impaired vascular repair. However, the clinical evidence remains heterogeneous. In HIV cohorts, there is evidence of proximal aortic remodeling and a clinically meaningful aneurysm burden across vascular foci. Within oncology cohorts, aggregate analyses generally do not support a uniform acceleration of aneurysm growth with malignancy-directed therapy, and the most thoracic aorta-specific evidence suggests minimal AscAA expansion without a detectable chemotherapy/radiotherapy association. In transplant and oncologic immunosuppression, proximal surgical and echocardiographic cohorts suggest negative thoracic aortic remodeling signals but do not consistently demonstrate an advanced thoracic aneurysm phenotype or worse postoperative outcomes, reinforcing that immunosuppression may contribute to aortic growth in constellation with other high-risk features, rather than as a primary driver. Accordingly, our review cannot yet support additions to established guideline-based management; instead, it identifies evidence gaps where future work may add to the current literature on the contribution of immunosuppression to thoracic aneurysm pathology. Thoracic segment-specific, longitudinal imaging studies with prospective ascertainment of thoracic endpoints beyond diameter alone (i.e., dissection, rupture, and operative indications), ideally paired with mechanism-linked disease phenotyping, will help to better define risk stratification and long-term management strategies of immunosuppressed patients with thoracic aortic aneurysms.

## Figures and Tables

**Table 1:** Summary of studies.

Study	Year	Study Period	Population	N	Comparator Group	Category	Immunotherapy Exposure Definition	Exposure	Aortic Segment(s) Assessed	Thoracic/Ascending Endpoint(s) and Definitions	Follow-Up
*Ostovar* [11]	2022	2006–2016	Adults undergoing surgery for proximal thoracic aortic aneurysm (root/ascending/arch).	224 (ImSup 88; NoImSup 136)	Prior cytostatic/immunosuppressive exposure vs. none	Oncologic	History of cytostatic chemotherapy and/or long-term systemic immunosuppressive therapy (incl. glucocorticoids and other immunosuppressants).	ImSup 88/224 (39.3%): chemotherapy 38/88 (43.18%); long-term systemic glucocorticoids 35/88 (39.77%); other immunosuppressants 29/88 (32.95%); overlap possible.	Aortic root; ascending aorta (at pulmonary artery level); aortic arch (between brachiocephalic and left carotid).	Pre-op diameters at 3 thoracic levels; post-op diameters at 1 year; perioperative complications; 30-day and 1-year mortality; long-term survival (ImSup vs. NoImSup: long-term 93.2% vs. 93.4%; *p* = 0.344).	1-year post-op imaging; survival follow-up duration not explicitly reported.
*Leopardi* [12]	2017	January 2010–June 2016	Patients with concomitant malignancy and aortic aneurysms (mixed segments).	19	Oncologic therapy vs. no oncologic therapy during observation	Oncologic	Receipt of oncologic therapy during aneurysm observation (agents variably reported; timing/dose not specified).	NR (subset treated; included 1 TAA).	Mixed aortic (AAA predominant; includes 1 TAA).	Aneurysm growth/rupture events during oncologic treatment vs. no treatment (reported mean sac growth 2.9 cm over 6 months in treated subset; 2 urgent repairs for rupture; non-treated subset reported no growth).	~6 months (reported in treated subset); overall follow-up not reported.
*Høgh* [13]	2021	COCOMO recruitment/CT: March 2015–December 2016	People living with HIV (PLWH) and age/sex-matched HIV− controls; contrast-enhanced CT screening.	594 PLWH; 1188 HIV− controls	Matched HIV− controls	Acquired immunodeficiency (HIV)	HIV serostatus (with HIV-related metrics captured, e.g., CD4 count/viral load/ART).	594/1782 (33.3%) PLWH	Thoracic and abdominal aorta (multiple CT levels).	Aortic aneurysm defined per ESC guidance (≥50% increase over expected); operational thresholds included ascending ≥ 45 mm, descending ≥ 40 mm, and abdominal ≥ 30 mm (plus suprarenal ≥ 30 mm).	Cross-sectional (baseline CT).
*Wang* [14]	2014	1 January 2000–31 December 2006 Taiwan NHIRD)	Nationwide SLE cohort with matched healthy controls.	15,209 SLE; 60,836 controls	Matched healthy controls	Autoimmune disease (SLE)	Administrative-code defined SLE; outcome ascertainment by ICD codes for aortic aneurysm/dissection.	SLE vs. control	Aortic aneurysm and aortic dissection (administrative coding; segment not specified).	Incident aortic aneurysm/dissection rates: SLE 4.26 vs. control 1.27 per 10,000 person-years; IRR 3.34 (95% CI 1.71–6.91).	Mean follow-up: SLE 5.09 years; controls 6.69 years.
*Minhas* [15]	2022	October 2017–January 2019 (TTE acquisition window)	Men with and without HIV; echocardiographic measures (MACS).	1164 (645 HIV+; 519 HIV−)	HIV− controls	Acquired immunodeficiency (HIV)	HIV serostatus plus HIV metrics and inflammatory biomarkers.	645/1164 (55.4%) HIV+	Aortic annulus, aortic root, sinotubular junction, ascending aorta (TTE).	Aortic root and ascending aorta size on transthoracic echocardiography; regression models linking HIV/inflammation metrics to proximal aortic dimensions.	Cross-sectional.
*Chehab* [16]	2022	2013–2019 (US National Inpatient Readmission Database)	Hospitalized adults with aortic aneurysm (AA) with vs. without HIV (ICD-coded).	1,905,837 AA hospitalizations; HIV+ 4416 (0.23%)	AA hospitalizations without HIV	Acquired immunodeficiency (HIV)	HIV diagnosis codes within NRD hospitalization records.	4416/1,905,837 HIV+	Aortic aneurysm (administrative coding; thoracic vs. non-thoracic coded).	Thoracic aneurysm prevalence and inpatient outcomes (rupture/dissection/repair/mortality) using ICD codes; 30-day readmission captured via NRD linkage.	Index hospitalization + 30-day readmission (NRD).
*Grønbæk* [17]	2023	COCOMO CT/biomarkers: March 2015–December 2016	PLWH with contrast-enhanced CT and biomarker profiling.	571 PLWH	Aneurysm vs. no aneurysm within PLWH cohort	Acquired immunodeficiency (HIV)	HIV serostatus; aneurysm status defined by CT thresholds; biomarkers of platelet/hemostatic/endothelial activation.	571/571 (100%) PLWH	Thoracic and abdominal aorta (multiple CT levels).	Presence of aortic aneurysm by CT (operational thresholds aligned with ESC guidance); associations with biomarkers (e.g., sCD40L, D-dimer, syndecan-1).	Cross-sectional.
*Kim* [18]	2021	2013–2018 (2 institutions)	HIV-positive patients with aneurysms; anatomic distribution and repairs.	104 patients; 129 aneurysms	Descriptive series	Acquired immunodeficiency (HIV)	HIV diagnosis with extracted HIV metrics (CD4/viral load/ART classes; AIDS history).	100% HIV+	Multiple vascular beds; includes ascending aorta and descending thoracic aorta (plus abdominal/iliac/cerebral/other).	Distribution of aneurysms by location (e.g., 53 ascending; 13 descending thoracic) and acute thoracic events (3 type A dissections; descending thoracic rupture among 7 total ruptures); repair types and 30-day outcomes.	30-day outcomes for repairs; selected longer follow-up reported by vascular bed (variable).
*Kurata* [19]	2011	Literature cases 1969–2008 (meta-analysis)	Reported cases of SLE-associated aortic aneurysm/dissection.	35 cases	Within-case comparison of thoracic vs. abdominal phenotypes	Autoimmune disease (SLE) ± therapy	SLE cases; steroid duration and atherosclerosis/vasculitis features abstracted from reports.	Variable (case-based)	Thoracic vs. abdominal aorta (case-based).	Phenotype patterns: inflammatory/medial degeneration pathway (thoracic-predominant) vs. steroid/atherosclerotic pathway (abdominal-predominant); rupture/dissection outcomes described.	Not reported (case reports).
*Zhang* [20]	2021	2000–2020	Single-center SLE cohort with aortic aneurysm (AA) cases and SLE controls.	1843 SLE; 16 AA cases (0.87%); comparator sample 160 SLE without AA	SLE without AA (randomly selected sample)	Autoimmune disease (SLE; treated)	Clinical SLE cohort; treatments and disease features extracted; AA defined by imaging/clinical records.	AA cases vs. non-AA SLE controls	Aortic aneurysm (thoracic and/or abdominal; mixed).	Prevalence and clinical features of AA in SLE; outcome analyses including survival (log-rank *p* = 0.03 for AA vs. non-AA).	Non-AA group: median follow-up 10 years; AA group follow-up not reported.
*Cron* [21]	2014	23 February 2000–6 October 2011	Abdominal-organ transplant recipients with aneurysms (identified via EMR).	3216 transplants; 127 aneurysm patients (including 22 TAA)	Descriptive	Transplant	Transplant status (immunosuppression implied; regimen-level details variably available).	127/3216 (3.9%) with aneurysm diagnosis	AAA and TAA (plus other aneurysm beds).	AAA ≥ 3.0 cm; TAA ≥ 3.75 cm; timing of detection (pre- vs. post-transplant) and rupture events; limited longitudinal growth subset (AAA pre- vs. post-transplant).	Variable; serial imaging subset only (no uniform follow-up reported).
*Obremska* [22]	2018	Not reported (cross-sectional echocardiograph cohort)	Kidney transplant recipients undergoing echocardiography; regimen comparison.	87 (mTORi 41; CNI 46)	mTOR inhibitor vs. calcineurin inhibitor regimen	Transplant	Maintenance regimen category (mTOR inhibitor vs. calcineurin inhibitor).	41/87 (47%) mTORi; 46/87 (53%) CNI	Aortic root and ascending aorta (echo).	Aortic root and ascending aortic diameters on echo; enlargement defined by observed vs. expected diameter; regression models included BSA and regimen exposure.	Cross-sectional.
*Maxwell* [23]	2021	2005–2017	Patients with small aortic aneurysms with and without malignancy/chemotherapy/radiation exposure.	Malignancy: 159 patients/172 aneurysms; No malignancy: 127/149	No malignancy	Oncologic	Concomitant malignancy with documented chemo and/or radiation; imaging before/during/after therapy (≥3 studies).	Active malignancy/therapy cohort vs. non-malignancy cohort	AAA, TAA, and thoracoabdominal aneurysms (multimodality imaging).	Median growth rates overall (0.12 vs. 0.12 cm/year); repairs/rupture; class-specific models (e.g., antimetabolites β = 0.170 for growth; topoisomerase inhibitors HR 2.694 for earlier repair).	Median follow-up 28.2 months (range 3.1–174.4).
*Martin* [24]	2015	January 2000–July 2011 (chemo cohort); January 2000–July 2007 (surveillance controls)	Patients with aortic aneurysm receiving cytotoxic chemotherapy vs. surveillance controls.	Chemo cohort: 91; surveillance controls: 354	No chemotherapy	Oncologic	Receipt of cytotoxic chemotherapy during aneurysm surveillance (agents, duration, and steroid co-use abstracted).	91 chemotherapy-exposed (steroid co-use 84/91, 92%)	Mixed aneurysm beds (AAA predominant; includes ascending, thoracic, thoracoabdominal, iliac).	Aneurysm growth rate (2.3 vs. 2.4 mm/year chemo vs. controls); catastrophic events and interventions during chemotherapy (includes ascending and thoracoabdominal cases).	Surveillance controls: mean follow-up 22 months; intervention subgroup: mean follow-up 26 ± 24 months.
*Becker von Rose* [25]	2023	1 January 2003–28 February 2021	Patients with concomitant malignancy and ascending aortic aneurysm (AscAA) identified via PACS CT reports.	151 (145 analyzable for growth; follow-up ≥ 6 months with ≥2 CTs)	Within-cohort comparisons by therapy exposure (chemo YES/NO; radiation YES/NO; combinations)	Oncologic	Tumor diagnosis and therapy overlapping aneurysm diagnosis/CT observation (chemo, radiation, surgery; combinations).	Chemotherapy 111/151 (73.5%); radiotherapy 75/151 (49.7%); surgery 93/151 (61.6%) (therapies overlap).	Ascending aorta (root/SOV, STJ, tubular ascending to innominate artery).	AscAA defined as transverse diameter > 40 mm; annual growth rate on serial CT (mean 0.18 ± 0.64 mm/year); no significant association with chemo/radiation/tumor entity.	Observation time mean 3.9 ± 3.2 years; CT follow-up requirement ≥ 6 months.

Abbreviations: **AA**: aortic aneurysm; **AAA**: abdominal aortic aneurysm; **AscAA**: ascending aortic aneurysm; **TAA**: thoracic aortic aneurysm; **AIDS**: acquired immunodeficiency syndrome; **ART**: antiretroviral therapy; **BSA**: body surface area; **CD4**: cluster of differentiation 4; **CI**: confidence interval; **CNI**: calcineurin inhibitor; **COCOMO**: Copenhagen Comorbidity in HIV Infection study; **CT**: computed tomography; **EMR**: electronic medical record; **ESC**: European Society of Cardiology; **HIV**: human immunodeficiency virus; **HR**: hazard ratio; **ICD**: International Classification of Diseases; **IRR**: incidence rate ratio; **MACS**: Multicenter AIDS Cohort Study; **mTORi**: mammalian target of rapamycin inhibitor; **NHIRD**: National Health Insurance Research Database; **NR**: not reported; **NRD**: National Inpatient Readmission Database; **PACS**: picture archiving and communication system; **PLWH**: people living with HIV; **SLE**: systemic lupus erythematosus; **SOV**: sinuses of Valsalva; **STJ**: sinotubular junction; **TTE**: transthoracic echocardiography; **sCD40L**: soluble CD40 ligand.

## Data Availability

No new data were created or analyzed in this study. Data sharing is not applicable to this article.

## Abbreviations

AAS: acute aortic syndrome; AA: aortic aneurysm; AAA: abdominal aortic aneurysm; AscAA: ascending aortic aneurysm; ARB: angiotensin receptor blocker; ART: antiretroviral therapy; BSA: body surface area; CI: confidence interval; CNI: calcineurin inhibitor; CT: computed tomography; CTA: computed tomography angiography; ECG: electrocardiogram; IMH: intramural hematoma; IRR: incidence rate ratio; MACS: Multicenter AIDS Cohort Study; mTORi: mammalian target of rapamycin inhibitor; NR: not reported; PAU: penetrating aortic ulcer; PLWH: people living with HIV; SLE: systemic lupus erythematosus; STJ: sinotubular junction; TAA: thoracic aortic aneurysm; TEE: transesophageal echocardiography; TTE: transthoracic echocardiography; VSMC: vascular smooth muscle cell.
